# Impact of Primary Tumor Location on Demographics, Resectability, Outcomes, and Quality of Life in Finnish Metastatic Colorectal Cancer Patients (Subgroup Analysis of the RAXO Study)

**DOI:** 10.3390/cancers16051052

**Published:** 2024-03-05

**Authors:** Sonja Aho, Emerik Osterlund, Ari Ristimäki, Lasse Nieminen, Jari Sundström, Markus J. Mäkinen, Teijo Kuopio, Soili Kytölä, Annika Ålgars, Raija Ristamäki, Eetu Heervä, Raija Kallio, Päivi Halonen, Leena-Maija Soveri, Arno Nordin, Aki Uutela, Tapio Salminen, Hanna Stedt, Annamarja Lamminmäki, Timo Muhonen, Juha Kononen, Bengt Glimelius, Helena Isoniemi, Juho T. Lehto, Kaisa Lehtomäki, Pia Osterlund

**Affiliations:** 1 Department of Oncology, Tays Cancer Centre, Tampere University Hospital, Elämänaukio 2, 33520 Tampere, Finland; sonja.aho@pirha.fi (S.A.); tapio.salminen@pirha.fi (T.S.); kaisa.lehtomaki@tuni.fi (K.L.); 2 Faculty of Medicine and Health Technology, Tampere University, Arvo Ylpön katu 23, 33520 Tampere, Finland; 3 TUNI Palliative Care Research Group, Faculty of Medicine and Health Technology, Tampere University, Arvo Ylpön katu 23, 33520 Tampere, Finland; juho.lehto@tuni.fi; 4 Palliative Care Centre, Tampere University Hospital, Elämänaukio 2, 33520 Tampere, Finland; 5 Department of Immunology, Genetics and Pathology, Uppsala University, 75185 Uppsala, Sweden; emerik.osterlund@igp.uu.se (E.O.); bengt.glimelius@igp.uu.se (B.G.); 6 Department of Transplantation and Liver Surgery, Helsinki University Hospital, Haartmaninkatu 4, 00290 Helsinki, Finland; arno.nordin@hus.fi (A.N.); aki.uutela@hus.fi (A.U.); helena.isoniemi@hus.fi (H.I.); 7 Department of Pathology, HUSLAB, HUS Diagnostic Center, Helsinki University Hospital, Haartmaninkatu 3, 00290 Helsinki, Finland; ari.ristimaki@hus.fi; 8 Applied Tumor Genomics Research Program, Research Programs Unit, Faculty of Medicine, University of Helsinki, Haartmaninkatu 8, 00290 Helsinki, Finland; 9 Department of Pathology, FIMLAB, Tampere University Hospital, Elämänaukio 2, 33520 Tampere, Finland; lasse.nieminen@fimlab.fi; 10 Department of Pathology, University of Tampere, Arvo Ylpön katu 23, 33520 Tampere, Finland; 11 Department of Pathology, Turku University Hospital, Kiinanmyllynkatu 4-8, 20520 Turku, Finland; jari.sundstrom@tyks.fi; 12 Institute of Biomedicine, University of Turku, Kiinanmyllynkatu 10, 20520 Turku, Finland; 13 Department of Pathology, Oulu University Hospital, Kajaanintie 50, 90220 Oulu, Finland; markus.makinen@oulu.fi; 14 Translational Medicine Research Unit, Department of Pathology, University of Oulu, Pentti Kaiteran katu 1, 90570 Oulu, Finland; 15 Medical Research Center Oulu, Pentti Kaiteran katu 1, 90570 Oulu, Finland; 16 Department of Pathology, Hospital Nova, Hoitajantie 3, 40620 Jyväskylä, Finland; teijo.kuopio@gmail.com; 17 Department of Biological and Environmental Science, University of Jyväskylä, Seminaarinkatu 15, 40014 Jyväskylän yliopisto, Finland; 18 Department of Genetics, HUSLAB, HUS Diagnostic Center, Helsinki University Hospital, Haartmaninkatu 3, 00290 Helsinki, Finland; soili.kytola@hus.fi; 19 Department of Genetics, University of Helsinki, Haartmaninkatu 8, 00290 Helsinki, Finland; 20 Department of Oncology, Turku University Hospital and University of Turku, Hämeentie 11, 20520 Turku, Finland; annika.algars@tyks.fi (A.Å.); rairis@utu.fi (R.R.); eetu.heerva@tyks.fi (E.H.); 21 Department of Oncology, Oulu University Hospital, Kajaanintie 50, 90220 Oulu, Finland; raija.kallio@ppshp.fi; 22 Department of Oncology, University of Oulu, Pentti Kaiteran katu 1, 90570 Oulu, Finland; 23 Department of Oncology, Helsinki University Hospital, Haartmaninkatu 4, 00290 Helsinki, Finland; paivi.halonen@hus.fi; 24 Department of Oncology, University of Helsinki, Haartmaninkatu 8, 00290 Helsinki, Finland; leena-maija.soveri@keusote.fi (L.-M.S.); timo.muhonen@mediexpert.fi (T.M.); 25 Home Care, Joint Municipal Authority for Health Care and Social Services in Keski-Uusimaa, Sairaalakatu 1, 05850 Hyvinkää, Finland; 26 Department of Surgery, University of Helsinki, Haartmaninkatu 8, 00290 Helsinki, Finland; 27 Department of Oncology, Kuopio University Hospital, Puijonlaaksontie 2, 70210 Kuopio, Finland; hanna.stedt@pshyvinvointialue.fi (H.S.); annamarja.lamminmaki@pshyvinvointialue.fi (A.L.); 28 Faculty of Health Sciences, University of Eastern Finland, Yliopistonranta 1A, 70210 Kuopio, Finland; 29 Department of Oncology, South Carelia Central Hospital, Valto Käkelän Katu 1, 53130 Lappeenranta, Finland; 30 Docrates Cancer Centre, Docrates Hospital, Saukonpaadenranta 2, 00180 Helsinki, Finland; juha.kononen@docrates.com; 31 Department of Oncology, Hospital Nova, Hoitajankatu 3, 40620 Jyväskylä, Finland; 32 Department of Gastrointestinal Oncology, Karolinska Universitetssjukhuset, Eugeniavägen 3, 17176 Solna, Sweden; 33 Department of Oncology/Pathology, Karolinska Institutet, Solnavägen 1, 17177 Solna, Sweden

**Keywords:** metastatic colorectal cancer, primary tumor location, resectability, metastasectomy, quality of life

## Abstract

**Simple Summary:**

The location of the primary tumor in the right colon, left colon, or rectum affects the efficacy of biological drugs used in the treatment of metastatic colorectal cancer, but how? We examined how the primary tumor location affects disease characteristics, treatability, quality of life, and outcome in a real-life study population of 1080 Finnish patients in the RAXO study. The primary tumor location correlates with the location of metastases, the frequency of gene mutations, how often metastases can be operated upon, long-term survival after curative surgery or palliative chemotherapy, and the quality of life during the disease trajectory. The primary tumor location is a helpful surrogate for clinicians working with metastatic colorectal cancer patients in estimating the clinical course of the disease. This study cannot identify the reasons for the associations, i.e., whether it is the primary location per se, the different mutations, or other reasons.

**Abstract:**

The primary tumor location (PTL) is associated with the phenotype, metastatic sites, mutations, and outcomes of metastatic colorectal cancer (mCRC) patients, but this has mostly been studied according to sidedness (right vs. left sided). We studied right colon vs. left colon vs. rectal PTL in a real-life study population (*n* = 1080). Health-related quality of life (HRQoL) was assessed multi-cross-sectionally with QLQ-C30, QLQ-CR29, EQ-5D, and 15D. A chi-square, Kaplan–Meier, and Cox regression were used to compare the groups. The PTL was in the right colon in 310 patients (29%), the left colon in 396 patients (37%), and the rectum in 375 patients (35%). The PTL was associated with distinct differences in metastatic sites during the disease trajectory. The resectability, conversion, and resection rates were lowest in the right colon, followed by the rectum, and were highest in the left colon. Overall survival was shortest for right colon compared with left colon or rectal PTL (median 21 vs. 35 vs. 36 months), with the same trends after metastasectomy or systemic therapy only. PTL also remained statistically significant in a multivariable model. The distribution of symptoms varied according to PTL, especially between the right colon (with general symptoms of metastases) and rectal PTL (with sexual- and bowel-related symptoms). mCRC, according to PTL, behaves differently regarding metastatic sites, resectability of the metastases, outcomes of treatment, and HRQoL.

## 1. Introduction

The primary tumor location (PTL) affects the phenotype, treatment alternatives, and prognosis of metastatic colorectal cancer (mCRC) [1]. The negative prognostic and predictive value of PTL for epidermal growth factor receptor (EGFR)-inhibitor treatment has led to renewed interest in the PTL [2,3]. In a large population-based SEER and national program of cancer registries material from the US, in patients with stage I–IV cancers, right colon primaries were present in 39%, left colon cancers in 24%, and rectal cancers in 30% [4].

The right side of the colon located proximal to the splenic flexure (caecum, ascending colon, and proximal two-thirds of the transverse colon), arises from the midgut during embryological development, while the left colon and rectum arise from the hindgut. Probably due to differences in the gut microbiota, the right side of the colon displays differences in its mucosal immunology [5]. Right-sided tumors are more frequently mucinous, are associated with an inflammatory response, and have a higher frequency of *BRAF*-V600E mutations (mt), deficient mismatch repair (dMMR)/microsatellite instability high (MSI-H), and hypermutated tumors [6,7]. Left-sided colon or rectal tumors more frequently have chromosomal alterations, amplification of EGFR and HER2 genes, and aberrant EGFR signaling. There are also variations between left colon and rectum, for example, regarding *KRAS* frequencies [6,7,8]. The location of metastases also differs notably, with right-sided cancers having more peritoneal carcinomatosis, left-sided colon cancers having more liver metastases, and rectal cancers having more lung metastases [9]. The surgical and radiation therapy approaches also differ for rectal and colon primaries. This means that right colon, left colon, and rectal PTLs can, at least in some respects, be considered as different diseases from a clinical perspective. 

Patients with an unresectable right-sided mCRC have worse overall survival (OS) and progression-free survival (PFS) rates, independent of the treatment regimens studied, compared to those with left-sided primaries [3,10,11,12,13]. PTL also influences the prognosis after a liver resection, with metachronous left-sided mCRCs having the best survival, and right-sided the worst [14,15]. The first-line combination of chemotherapy with EGFR-inhibitors has clearly benefitted patients with left-sided tumors, whereas patients with right-sided tumors have derived limited benefits in most studies, but, on the contrary, seem to benefit from the addition of bevacizumab [2,3,10,16].

Health-related quality of life (HRQoL) and maintenance of functionality are highly important to patients [17,18]. Thus, in addition to classical clinical trial endpoints, such as survival, measuring functional, social, and emotional parameters of HRQoL is especially valuable in studies exploring treatment decisions, to maximize resectability, survival, and palliation [19]. In phase III studies of systemic treatments, a HRQoL measurement is recommended but far from always reported, and real-life data for HRQoL during and after systemic treatment are scarce [20,21,22]. Metastasectomies have been used increasingly and improve survival significantly and, thus, focus on long-term adverse events is of great importance. There are HRQoL data after a single organ metastasectomy, but very scarce data for multisite metastasectomies [21,23,24,25,26,27]. The HRQoL is also affected by the surgery for the primary tumor and the radiotherapy. To the best of our knowledge, there are no data on HRQoL in patients treated for multisite metastatic disease according to the PTL. 

Our aim was to assess the impact of right colon, left colon, or rectal PTLs on the demographics, resectability, and outcomes after metastasectomy and/or local ablative therapy (LAT), systemic therapy, or best supportive care (BSC) in a real-life Finnish study population of treatable patients with comprehensive clinical data and molecular testing. A secondary aim was to study HRQoL divided by the PTL during different disease phases. 

## 2. Materials and Methods

The RAXO study included 1086 mCRC patients between 2012 and 2018 [28]. Inclusion criteria were, in brief, patients eligible for first-line systemic therapy, an age of over 18 years, and a histologically confirmed CRC with distant metastases, or a locally advanced primary tumor not curatively treatable (but, in the end, no locally advanced patients were included, only metastatic). The resectability assessment, definitions of resectability, and data collection have been explained in a previous paper by Osterlund et al. [28].

The patients were treated according to local clinical guidelines, based on the ESMO and NCCN guidelines [29,30,31,32]. 

The molecular testing and testing for dMMR were described in an earlier study [33].

Clinical trial identification for the RAXO study is NCT01531595 https://classic.clinicaltrials.gov/ct2/show/NCT01531621, accessed on 26 February 2024 and EudraCT 2011-003137-33 https://eudract.ema.europa.eu/results-web/, accessed on 26 February 2024. Ethical permission for the study was obtained by the Ethical Board at the Helsinki University Hospital (number 242/13/03/02/2011 and HUS/1288/2016). The study was conducted in accordance with the Declaration of Helsinki. All patients gave their informed consent to participation to the prospective study and separately to the QoL study.

**Figure 1:**
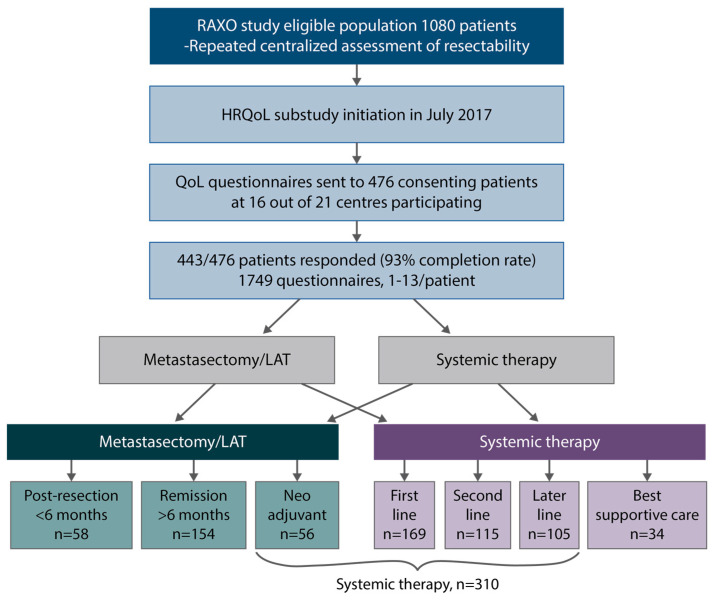
Study design, patient flow, health-related quality of life (HRQoL) questionnaires, post-resection (within 6 months after metastasectomy and/or local ablative therapy (LAT) including adjuvant-like treatment), remission (more than 6 months after metastasectomy and/or LAT), systemic therapy (mean of neoadjuvant/conversion, first-, second- and later-line), best supportive care (after ending active cancer treatment).

HRQoL was evaluated using four different HRQoL measures: the generic 15D [34] and EQ-5D-3L (index score and visual analogue scale [VAS]) [35], which produce both index and profile data, and the cancer-specific EORTC QLQ-C30 [36] and colorectal cancer-specific QLQ-CR29 [37], of which QLQ-C30 produces both an index (global health status—GHS) and profile measures, and QLQ-CR29 colorectal cancer-specific profile measures. The HRQoL assessments were previously described in detail [21]. The HRQoL data were collected multi-cross-sectionally (1–13 times) and analyzed according to disease phase (Figure 1). The questionnaires were given to the patients at the hospital or sent out by mail. The patients were instructed to fill out the questionnaires just before a response evaluation and/or a doctor’s appointment. The time points were, thus, not treatment-phase-dependent or scheduled to baseline, at certain timepoints during a treatment phase, or after progression. Four disease phases were used: post-resection, remission, systemic treatment, and BSC. The phases of curative treatment were defined as post-resection during the first 6 months after metastasectomy and/or LAT including any adjuvant therapy, and the remission phase started if the patient had been disease-free for more than 6 months from the last metastasectomy and/or LAT or had a complete response to the systemic therapy for more than 6 months. The systemic treatment phase included both non-curative systemic therapy in one or several lines that was given with the goal of life-prolongation and palliation but also neoadjuvant or conversion treatment that was given before metastasectomy and/or LAT. These two situations were combined to one group as it is not possible to know upfront if the treatment will result in a potentially curative metastasectomy/LAT or palliation [21]. The BSC phase was the time after ending active cancer treatment for mCRC (no patient was in the BSC only group since all patients should be treatable).

Results are presented as proportions, median with range or mean values with standard deviations or 95% confidence intervals (CI). Proportions between the three PTL groups and all demographic alternatives with percentage presented were compared using crosstabs. All comparisons were performed with the non-parametric Mann–Whitney or Kruskal–Wallis tests. Minimal clinically important difference (MID) was used with cut-offs as described in [21]. The reverse Kaplan–Meier method was used for estimation of median follow-up time. OS was estimated using Kaplan–Meier; it was calculated from time of mCRC diagnosis to death by any reason or censored if alive at last follow-up (7 October 2020). PFS was estimated from time of first-line systemic therapy initiation to progression or censored if no progression was noted at cut-off dates. Relapse-free survival (RFS) was calculated from first metastasectomy or LAT to relapse, death, or censored at last date of follow-up, and non-radical resection or second organ not resected denoted 0 months. OS and PFS were compared using Cox regression with 95% CI. A multivariable cox regression model adjusting for clinically meaningful variables was also fitted. Two-sided *p*-values < 0.05 and 95% CIs not crossing 1.00 were considered statistically significant. All analyses were performed using SPSS statistics version 28 or 29, IBM corporation, Armonk, NY, USA. 

## 3. Results

### 3.1. Baseline Demographics

The total number of patients was 1080, as multiple colorectal primaries were present in 6 cases omitted from further analysis in this sub-study. The PTL was right colon in 310 (29%), left colon in 396 (37%), and rectum in 374 (35%) (Table 1).

Patients with right colon tumors were more often over 70-years-old and females than patients with left colon or rectal PTLs. Right colon tumors more often had mucinous or signet cell histology and high-grade tumors than left colon or rectal primaries. Anemia was more common among right colon patients compared with left colon and rectum, but no other laboratory parameters, including CEA, differed according to PTL (Table S1).

Of the 1080 patients, 833 (77%) had their primary tumor resected. Surgical procedures for primary tumors are presented in Table 1. Primary tumors in the right or left colon were resected more often than the rectal tumors. 

**Figure 2:**
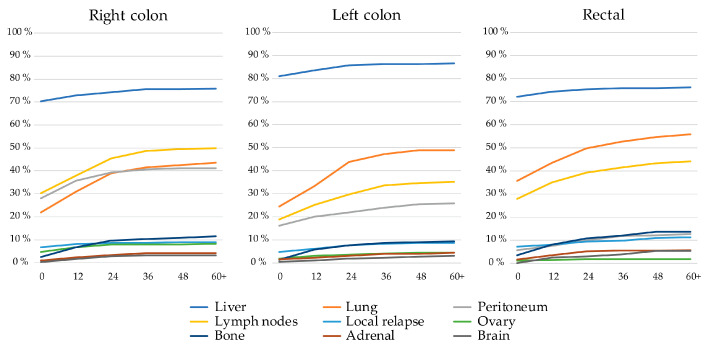
Frequency of metastatic sites of the nine most common metastatic sites at baseline and during disease trajectory (presented to 60+ months) divided by primary tumor location.

Patients with left colon tumors more often had liver metastases, both at baseline and during trajectory, than patients with right colon or rectal primaries (Figure 2). Rectal PTL was associated with a higher prevalence of lung metastases, almost doubling during trajectory, compared with patients with right colon and left colon primaries. Right colon PTL was associated with a higher prevalence of peritoneal, distant lymph node, and ovarian metastases compared to patients with left-sided colon and rectal PTLs. Liver metastases were mostly already present at baseline whereas lung, distant lymph node, and peritoneal metastases became more common over time, as did rarer metastatic sites such as bone, adrenal, or brain.

Of the included patients, 93% were adequately tested for *RAS* and *BRAF*-V600E mutations in clinical routine. *BRAF*-V600E and/or *NRAS* were missing in 59 patients (denoted (*K*)*RAS*) before the ESMO recommendation of extended testing in 2016 (Table 1). *RAS* & *BRAF* wild type (wt) status was more uncommon among the right colon PTL than in left colon or rectal PTLs. *RAS* mt were more common in rectal and right colon PTLs than in left colon PTL. Right colon PTL was associated with a higher proportion of *BRAF*-V600E mt compared with left colon or rectal PTLs. 

MMR testing was performed in 39% of the patients (Table 1). dMMR was more common in right colon PTL compared with left colon, with none identified in rectal PTL.

### 3.2. Resectability, Resections, and LAT

**Figure 3:**
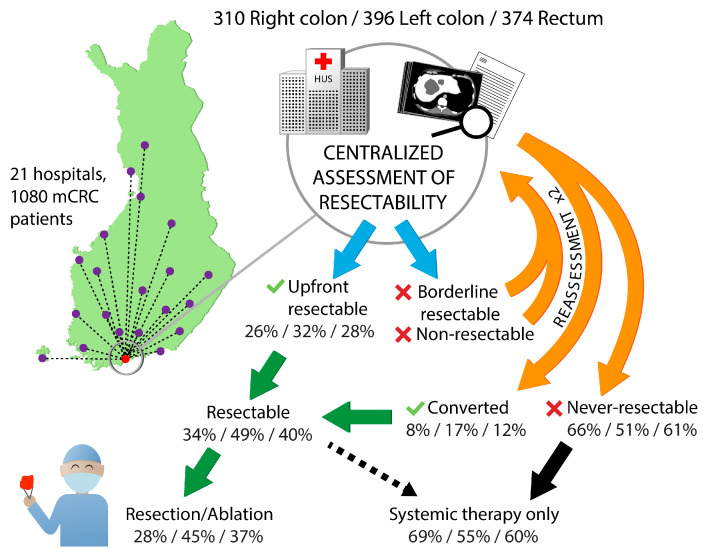
Technical resectability assessment at the tertiary center in Helsinki and the upfront resectable and converted proportions, proportions that underwent resection or ablation according to primary tumor location.

Technical resectability of metastases was centrally assessed for all patients (Figure 3). For patients with upfront borderline and non-resectable metastases, re-assessment was performed after 2–3 months and after 4–5 months of systemic therapy. 

Patients with right colon or rectal primaries had non-resectable metastases upfront more often compared with left colon primaries (60% vs. 58% vs. 48%), and after conversion therapy they remained never-resectable more often (Table 2).

Multisite and multiple metastasectomies were performed in 37% of the patients, with single site metastatic disease having a metastasectomy and/or LAT in 53% of patients and multiple metastatic site patients in 18%, respectively (Table 2). Mean number of metastasectomies/LAT per patient with a procedure was 1.6 in right colon PTL, 1.5 in left colon PTL, and 1.7 in rectal PTL. Resections and/or LAT were less frequently performed in patients with right colon PTL compared with left colon and rectum (28% vs. 45% vs. 37%), and, as a consequence they received ‘systemic therapy only’ more often (69% vs. 55% vs. 60%). 

Liver resections were performed most often in left colon PTL (Table 2). Of patients with baseline liver metastases or liver-only disease, metastasectomy and/or LAT was performed in 46% and 50%, respectively, compared with 26% and 24% in right colon PTL, and 39% and 36% of rectal PTL patients, respectively (*p* < 0.001). 

Lung resections were most often performed in rectal PTL (Table 2), with lung resections or LAT performed in 17% of the patients with baseline lung metastases and in 38% of patients with lung-only disease, compared with 9% and 40% in right colon PTL, and 12% and 50% in left colon PTL, respectively.

Cytoreductive surgery, distant lymphadenectomy, gynecologic resection, or subcutaneous resections were performed more often in right colon PTL. 

### 3.3. Treatments

Systemic therapy was given to 97% (1052/1080) of the patients, either as neoadjuvant/conversion/adjuvant or as non-curative treatment. The maximum number of treatment lines in a patient was seven. 

In first-line treatment, patients with right colon PTL were more often treated with bevacizumab-containing regimens compared to patients with left colon or rectal PTLs (66% vs. 54% vs. 56%, *p* = 0.005; Table 3), and on the contrary, less often with EGFR-inhibitors (5% vs. 18% vs. 17%, *p* < 0.001). EGFR-inhibitors were also less common in the *RAS* & *BRAF* wt subgroup (*n* = 354) in patients with right colon PTL (13% vs. 39% vs. 40%, *p* < 0.001).

During all lines of systemic therapy, patients with right colon PTL received VEGF-inhibitors (bevacizumab or aflibercept) as often as left colon or rectal PTL patients (76% vs. 70% vs. 71%, *p* = 0.176), whereas EFGR-inhibitors were given less often (15% vs. 38% vs. 33%, *p* < 0.001, in all patients; and 46% vs. 77% vs. 72%, *p* < 0.001, in the *RAS* & *BRAF* wt subgroup).

Good treatment response (i.e., partial or complete response, and no evidence of disease with metastasectomy and/or LAT) to first-line treatment was seen less often in right colon PTL compared with left colon and rectal PTLs (54% vs. 67% vs. 64%, *p* < 0.001; Table 3), and progressive disease as best response more often (16% vs. 6% vs. 8%, *p* < 0.001).

### 3.4. Overall Survival and Progression-Free Survival

**Figure 4:**
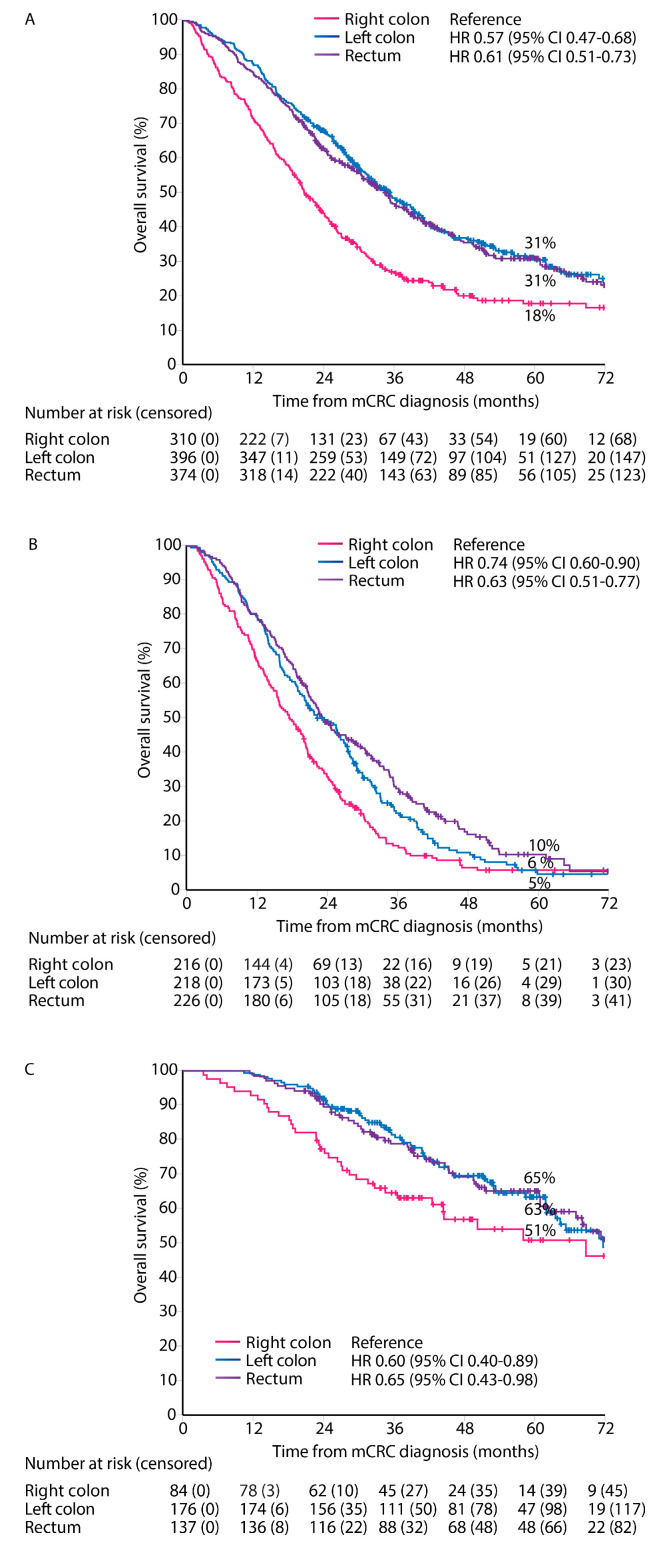
Overall survival according to primary tumor location. All patients (**A**), systemic therapy only (**B**), and metastasectomy and/or LAT (**C**).

Median follow-up time was 57 months (95% CI 54–60). OS from diagnosis of mCRC irrespective of treatment given was worse in right colon PTL compared with left colon and rectal PTLs (median OS 21 vs. 35 vs. 35 months, Figure 4). OS stratified by treatment group showed similar associations with PTL for ‘systemic therapy only’ (median OS 18 vs. 22 vs. 23 months), and ‘metastasectomy and/or LAT’ (median OS 69 vs. 72 vs. 73 months), but not for the ‘best supportive care only’ group (median 2 vs. 2. vs. 3 months, respectively).

**Figure 5:**
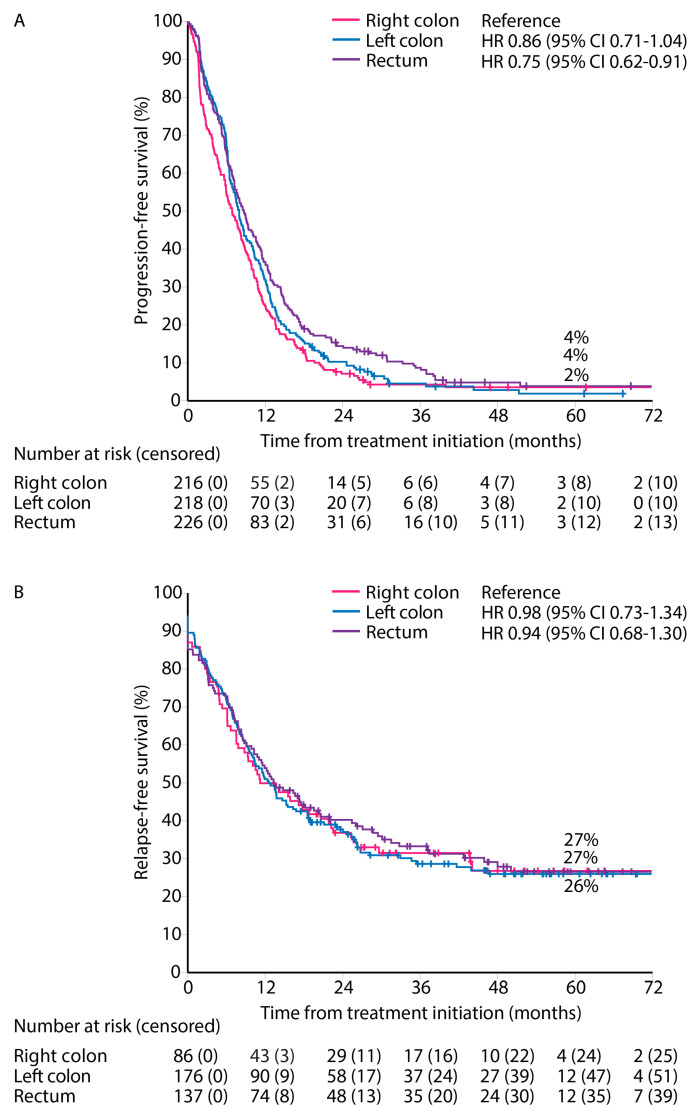
Progression-free survival for ‘systemic therapy only’ (**A**), and relapse-free survival after first metastasectomy and/or local ablative therapy according to primary tumor location (**B**).

PFS for first-line treatment for ‘systemic therapy only’ patients showed a worse PFS for right and left colon PTLs compared with rectal PTL (median PFS 7.0 vs. 7.6 vs. 8.3 months, Figure 5A).

RFS from first metastasectomy and/or LAT was similar for all PTLs (median RFS 11.2 vs. 12.6 vs. 13.4 months) and 5-year RFS rates (27% vs. 27% vs. 26%) for right colon, left colon, and rectal PTLs, respectively (Figure 5B). 

### 3.5. Multivariable Model for OS

OS was also impaired in right colon PTL compared with left colon and rectal PTLs in a multivariable model adjusted for age, number of metastatic sites, ECOG performance status, treatment modalities, and molecular pathology (HR reference, 0.77 [95% CI 0.63–0.93], 0.64 [0.53–0.78], Table 4).

Since treatment modality is not a “true” baseline factor, a second model using baseline resectability was constructed (Table S2). The results did not change substantially and the PTL was still statistically significant. 

### 3.6. HRQoL and PTL in Different Treatment Phases

HRQoL questionnaires were answered by 443 patients (1–13 questionnaires per patient), with 1749 questionnaires in total. In the post-resection phase, i.e., within 6 months from resection including adjuvant therapy, 58 patients responded, and during the remission phase, without relapse more than 6 months after metastasectomy/LAT, 154 patients responded. In the systemic treatment phase, where half of the patients were treated with curative neoadjuvant/conversion intent and half with non-curative intent, 310 patients answered and in the best supportive care phase, after stopping cancer treatment, 34 patients answered (Figure 1). 

Mean and SD values for the QoL indexes are presented in Table S3. The index scores for EQ-5D according to the three PTLs were 0.76–0.90 in the post-resection phase and 0.87–0.88 in the remission phase. 

When comparing right colon to left colon, an MID for worse index measures as 15D, EQ-5D, and GHS (the higher the better) was noted for the BSC phase, but no other MIDs or statistically significant differences were noted for the post-resection, remission, or systemic treatment phases (Table 5).

In a comparison between the right colon vs. rectal PTL, MIDs for 15D, EQ-5D, VAS, and GHS in the post-resection phase were noted; additionally, for 15D in the remission phase, and for GHS in the BSC phase (Table 5). Only the difference in VAS was statistically significant in the post-resection phase.

There were some minimal clinically important differences (Δ |0.05| or more) in 15D dimensions between the PTLs (Figure S1). 

In the post-resection phase, patients with right colon PTL scored better than those with left colon or rectal PTLs for excretion, discomfort, and symptoms, and worse for regarding sleeping, breathing, distress, and mental function. 

In the remission phase, patients with right colon PTL showed better MID compared with the left colon or rectal PTLs for sexual activity and excretion. For systemic treatment, those with right colon PTL showed better MIDs for sexual activity and worse for sleeping. In the BSC phase, right colon patients compared with rectal PTL scored better for breathing, excretion, usual activities, and mental function. 

### 3.7. QLQ-C30 and QLQ-CR29 Symptom Scales

Symptom burden (the lower the better, range 0–2600, MID ≥ |26 × 5 = 130|) is the individual sum of the means for the 26 different symptom scales of the QLQ-C30 and QLQ-CR29 questionnaires. The sum of 10 functioning scales (the higher the better, range 0–1000, MID ≥ |10 × 5 = 50|) from QLQ-C30 and QLQ-CR29 were also calculated. 

**Figure 6:**
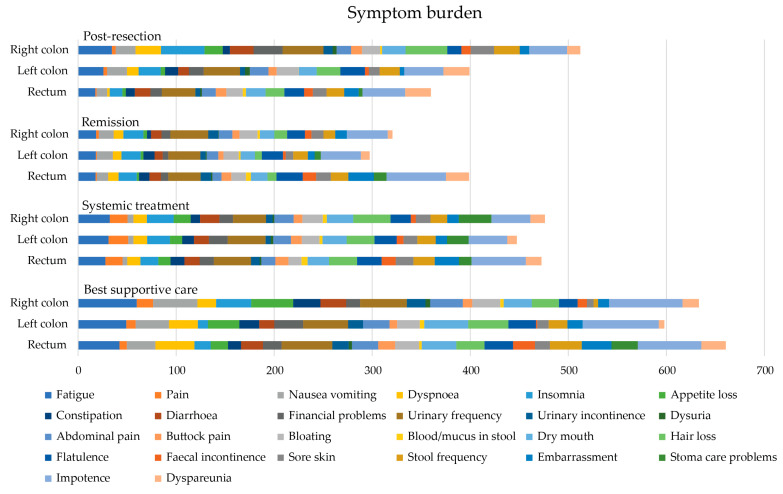
Symptom burden as sum of 26 symptom scales (0 no symptoms to 100 most symptoms, theoretical maximum 2600) measured with the QLQ-C30 and QLQ-CR29 during different treatment phases according to primary tumor location.

In the post-resection phase, patients with right colon PTL had a higher symptom burden and lower functioning scale sum, compared with those with left colon and rectal PTLs with a difference of 142 and −104, respectively, reaching the MID level but not statistical significance (Table 5 and Figure 6). In the post-resection phase, fatigue, dyspnea, insomnia, diarrhea, appetite loss, hair loss, sore skin, and stool frequency were clinically significantly (MID > 5) worse in patients with right colon PTL than in left colon or rectal PTLs. Symptoms more common in rectal PTL than in right colon and/or left colon PTLs were flatulence, embarrassment, and dyspareunia. 

**Figure 7:**
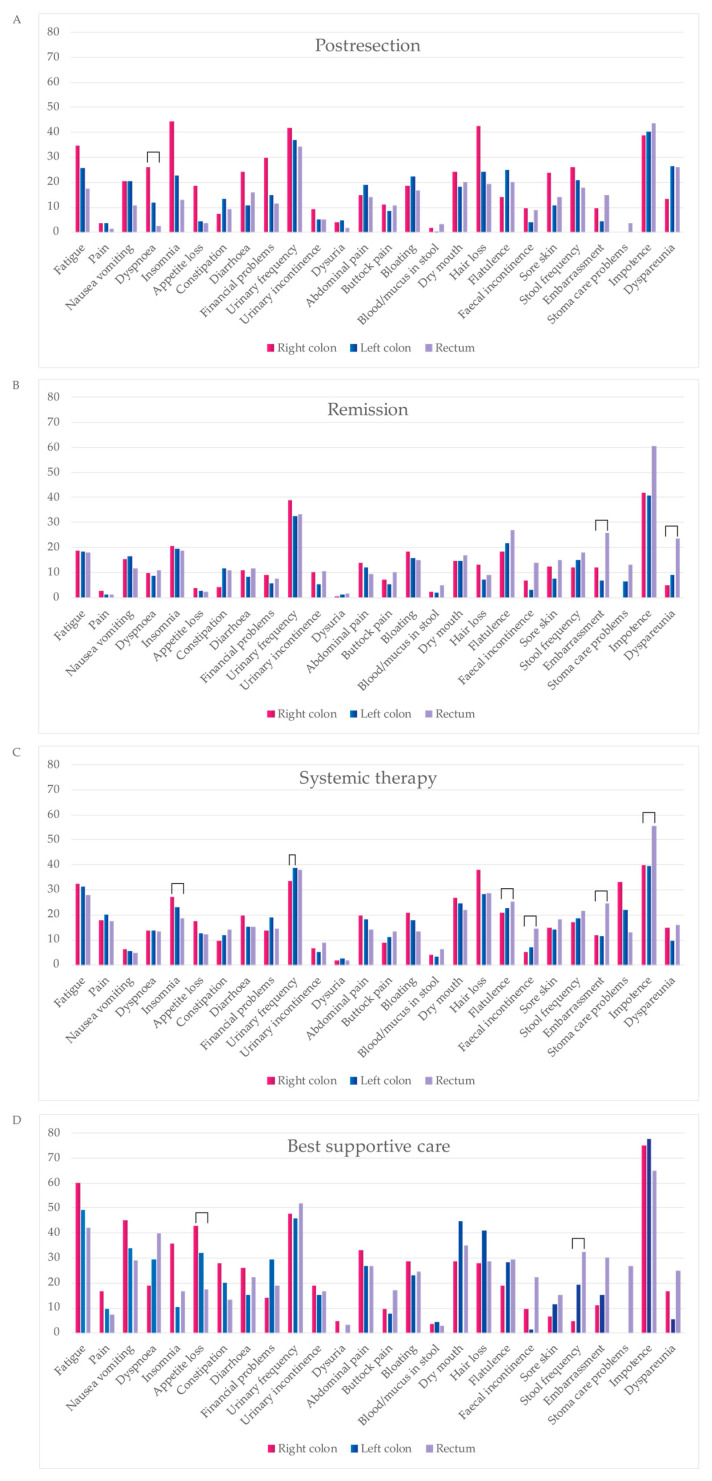
Comparison of symptom scales from QLQC30 during four treatment phases: post-resection ((**A**), within 6 months after metastasectomy and/or local ablative therapy (LAT) including adjuvant-like treatment), remission ((**B**), more than 6 months after metastasectomy and/or LAT), systemic therapy ((**C**), mean of neoadjuvant/conversion, first-, second- and later-line), best supportive care ((**D**), after ending active cancer treatment). Statistically significant differences between primary tumor locations are marked with brackets.

In the remission phase, symptom burden was numerically higher in patients with a rectal primary tumor compared to the right colon or left colon (Table 5). The symptoms that affected patient’s HRQoL after metastasectomy the most, regardless of PTL, were principally bowel- and sexuality-related (Figure 7). More symptoms in patients with rectal PTL compared with right and/or left colon were seen for constipation, flatulence, fecal incontinence, stool frequency, embarrassment, stoma care problems, impotence, and dyspareunia. 

In the systemic treatment phase, the primary tumor location did not statistically or clinically significantly affect the symptom burden (Table 5). During systemic treatment, stoma care problems were more common in patients with primaries in the right colon compared to the left and rectal PTLs (Figure 7). Instead, impotence and embarrassment were more common in patients with rectal PTL compared with the right or left colon PTLs.

For the BSC phase, no statistically significant differences between different PTLs for symptom burden were noted. The functioning scale sum was lower for rectal PTL compared with right and left colon PTLs (Table 5). In BSC, patients with a right colon PTLhad more fatigue, pain, insomnia, and appetite loss compared to left and rectal primary tumors (Figure 7). Patients with a left colon PTL had more dry mouth and hair loss compared with a right colon and rectal PTLs. Patients with a rectal PTL complained more often about dyspnea, stool frequency, fecal incontinence, embarrassment, stoma care problems, and impotence compared to right or left colon PTLs. 

## 4. Discussion

We have previously reported that by maximizing metastasectomies, it is possible to achieve excellent survival with maintained HRQoL in mCRC patients [21,28]. We now add that this is seen in all three PTLs. Patients with right colon PTL, associated with several negative predictive and prognostic factors, tended to do worse regarding resectability, conversions, resections, systemic treatment, and outcomes than those with the left colon PTL and more in line with the rectal PTL. There were also clinically important differences between left colon and rectal PTLs, and it may not always be accurate to lump them together as life-sided. Distribution of symptoms rendered typical for CRC and mCRC, nonetheless, varied according to the three PTLs, which has rarely been reported in the literature. HRQoL indexes were worse for right colon vs. left colon or rectal PTLs in post-resection and best supportive care phases, which has never been reported before. 

### 4.1. Resectability and Resections

To the best of our knowledge, resectability of mCRC metastases according to three PTL groups has not been reported before. Upfront resectability was slightly higher for left colon PTL compared with right colon and rectal PTLs (32% vs. 26% vs. 28%). However, a major difference is seen in the proportion of unconvertable and never-resectable, which was 66% among the right colon PTL compared with 51% for left colon and in between, 61%, for the rectal PTL. Thus, mCRC patients with a right colon PTL are also less often treated with curative intent metastasectomy and/or LAT (27%), compared with a left colon (44%) or rectal (37%) PTL. This is in line with differences noted in a population-based cohort of synchronous mCRC cases (11% vs. 13% vs. 16%) [9], or in the Cairo5 study with borderline or non-resectable liver metastases with resection rates of 58% in left-sided *RAS* & *BRAF* wt and 37% and 51% for right-sided and/or *RAS*/*BRAF* mt [38]. 

Right colon PTLs were associated with peritoneal, distant lymph node, and ovarian metastases, in line with previous findings [9]. Since these metastatic sites, as opposed to the liver and lungs, are rarely resectable, this can at least partly explain the differences in resectability and resection rates. Cytoreductive surgery was for this reason also performed more often in a right colon PTL. Liver metastases and liver-only were most common in left colon PTLs, in line with the findings in a Dutch study [9]. When liver metastases are present, they are more often synchronous, multiple, and affect more liver segments in a right colon PTL, compared with left colon or rectal PTLs [39], reflected in our liver-only patients who were resected in 24% of right colon PTL patients, compared with 50% of left colon, and 36% of rectal PTL patients. Further, a right colon PTL is more often *BRAF* mt and MSI-H, two groups associated with a more rapid clinical course, poorer performance, being another reason for fewer metastasectomies [40,41]. Patients with a rectal PTL had more lung metastases than right or left colon PTLs, in line with previously published reports [42], further adding to the difference in resectability and resection rates (9% in patients with baseline lung metastases and a right colon PTL vs. 12% in a left colon PTL vs. 17% in a rectal PTL). Metastases of right colon PTLs are thus more often unresectable at baseline and they also stay never-resectable more often than metastases in patients with left colon or rectal PTLs. Further, they more often have an unfavorable biology being less responsive to conventional chemotherapy. Treatments, thus, more often become non-curative systemic therapy. The lower conversion rate probably mimics the lower response rates seen in right colon PTL receiving systemic therapy whether with or without EGFR- or VEGF-inhibitors [2]; lower response rates were also seen here. The increasing incidence of lung, peritoneal, distant lymph node, and brain metastases during trajectory highlights a need for re-resections and multisite resections. An ambition of the RAXO study was to maximize resection rates and multiple resections also were more common (mean 1.5 resections per metastasectomized in right colon, 1.6 in left colon, and 1.7 in rectal PTL patients) in this study than in other studies. 

Right colon PTL was, as discussed above, associated with a higher incidence of *BRAF* mt and dMMR, and also high *RAS* mt rates, which were associated with lower metastasectomy rates [7,43,44]. This is not unexpected, since *BRAF* mt patients often have metastases in organs considered unresectable, such as the peritoneum and distant lymph nodes, while *RAS* mt patients have metastases in lungs and brain. It has been suggested that *BRAF* mt could be the genetic alteration that is responsible for differences in metastatic sites between the right colon and left colon PTLs [7]. 

The noted differences in patient demographics, such as female sex, older age, and high grade tumors, for a right colon PTL in this study are in line with previously published studies [45], and may also explain the lower metastasectomy rates.

### 4.2. Survival, Prognosis, and Predictive Factors

Due to poor prognostic features such as *BRAF* mt, dMMR, adverse metastatic sites, mucinous and signet cell tumor histology, high grade, female sex and older age, comorbidities, etc., associated with a right colon PTL, patients with a right colon PTL have a worse outcome as compared to left colon and/or rectal PTLs [9,45,46], in line with our findings. Earlier disease stages show similar differences in demographics between right colon and left colon PTLs as in mCRC, but there are no clinically meaningful differences in survival or recurrence [45,47,48]. This implies that the factors that drive CRC recurrence are, at least in part, distinct from those influencing survival in mCRC. 

OS and PFS for all patients, regardless of treatment and in systemic therapy, are generally inferior for right-sided compared with left-sided colorectal cancer [2,3,9,10,46], as also shown in our study. Previous studies have reported that a right colon PTL is associated with a lack of benefit when treated with EGFR-inhibitors; additionally, when the tumor is *RAS* wt [2,3,10] or *RAS* & *BRAF* wt and pMMR [49]. On the contrary, numerical PFS and OS benefit from the addition of bevacizumab is seen in a right colon PTL [3,10,50]. An OS benefit from the addition of an EGFR-inhibitor in left-sided *RAS* and/or *BRAF* wt patients has been seen in most studies, apart from Cairo5 [2,3,38,50]. Also, there are studies showing worse survival for rectal PTLs compared to left colon PTLs when treated with an EGRF-inhibitor [51,52]. 

A Dutch study showed impaired OS for patients with any metastasectomy or systemic therapy only in the right colon PTL subgroup compared with left colon and rectal PTLs [9]. This survival difference is also shown in the liver resection subgroup of that study (no OS-rates given) [9] and in an Austrian patient series (5-year OS rates approximately 30% for right-sided vs. 42% for left-sided) [15]. The Cairo5 study showed approximately 30+% 5-year OS rates in the right-sided or *RAS*/*BRAF* mt group and 40+% in the left-sided *RAS* & *BRAF* wt group [38]. This is in line with our findings of 5-year OS rates 51% vs. 63% vs. 65%, respectively, after any metastasectomy for a right colon PTL compared with left colon and rectal PTLs, with the caveat that survival after relapse is shorter in the right colon PTL. 

### 4.3. Health-Related Quality of Life

HRQoL after metastasectomy in mCRC has rarely been reported, and mostly for single-site metastasectomies [24,25,26,27], with data for multisite and multiple CRC metastasectomies available only from this study and a Canadian study [21,23]. To our knowledge, there are no previous data presented in this patient population divided according to PTL. 

As shown earlier, index measures, expressing the QoL with one number, without any profile or symptom measures, report preserved global HRQoL despite receiving more intense treatment, generally meaning more adverse events [53,54,55]. In line with this, this study shows more pronounced differences in symptom scores than in index scores. However, index scores (15D, EQ-5D, and GHS) showed worse HRQoL in patients with right colon PTL after metastasectomy compared to rectal PTL and worse HRQoL in BSC phase compared to left colon PTL. Patients with metastatic spread in the liver mostly recover quickly after modern liver surgery [56,57]. The fact that patients with right colon PTL have proportionally fewer liver and lung metastasectomies and more cytoreductive surgery could contribute to this HRQoL difference seen after metastasectomy. 

The impact of the PTL on HRQoL in mCRC from a surgical point of view focuses on the question whether and when the primary tumor should be resected or not. Surgery may improve QoL, especially with symptomatic primaries, but without apparent survival benefit in asymptomatic primaries [58,59]. Studies unquestionably show that surgery of stage I-III rectal tumors is an important driver of poor HRQoL in CRC survivors [60,61,62,63]. 

In this study, symptoms impairing HRQoL of metastatic rectal cancer patients were related to bowel function or sexuality. Symptoms as embarrassment, dyspareunia, impotence, low sexual interest, stoma care problems, fecal incontinence, stool frequency, constipation, flatulence, etc., bothered the rectal PTL patients during all treatment phases. All these symptoms have been generally described in mCRC patients, but not confined to rectal PTL [64,65,66]. 

Patients with a right colon PTL, compared with left colon and/or rectal PTLs, more often had symptoms like dyspnea, insomnia, hair loss, or loss of appetite. These, along with fatigue and insomnia, have been the most common complaints previously published but again without differentiation according to PTL [67,68,69,70]. These symptoms of a right colon PTL may reflect the metastatic spread in the peritoneal cavity and lymph nodes and may, especially in the postresection phase, be caused by extensive cytoreductive surgery. 

During the whole treatment trajectory, patients with a left colon PTL seemed to report less severe symptoms than those with right colon or rectal PTLs. 

As observed previously, the most symptoms were reported during the BSC phase but no studies have reported symptoms according to PTL [66,71,72].

The symptom burden is caused by the cancer itself and by the treatments. Awareness of long-term adverse events with surgical, radiotherapy, and systemic treatment need to be minimized with individualized treatment planning in multi-disciplinary teams, and patient preferences kept in mind with shared decision making. To capture all symptoms causing shame, like sexual complaints, urinary frequency, bowel function, and stoma care problems, these themes need to be actively discussed with patients, with a special emphasis on rectal PTL patients. Meanwhile, patients with a right colon PTL need support especially after metastasectomy and during systemic treatment. This emphasizes the need for survivorship programs [73,74]. 

### 4.4. Strengths and Limitations

The strengths of this study include its prospective setting with a large national real-life population, extensive and complete patient-level details, high rate of *RAS* and *BRAF* testing, and inclusion of patients with multiple and multisite metastases. An additional strength of the study is the use of multiple HRQoL questionnaires and high questionnaire completion rate of 93%, in comparison with 73–91% for CRC patients in other cross-sectional studies [75,76]. We also captured HRQoL data throughout the disease trajectory. 

An obvious limitation of this study is the observational design without randomization. Nevertheless, the long-term observational nature with a high number of patients has allowed us to describe the clinical behavior, treatments, and outcomes in detail. Another limitation is that MMR testing was not performed in the early days of inclusion. Central assessment without full knowledge of the patients’ condition may be criticized but provided a good estimate of technical resectability. A major limitation is that HRQoL questionnaires were recorded multi-cross-sectionally and not longitudinally at prespecified timepoints. The HRQoL sub-study started in 2017 when the RAXO study per se had been ongoing since 2012. This led to a risk of guarantee-time bias. The BSC phase describes the HRQoL after failing intensive treatment and not those receiving ‘BSC only’. 

## 5. Conclusions

mCRC behaves differently according to the primary tumor location regarding metastatic sites, resectability of metastases, outcomes of treatment, and quality of life. Patients with right colon primaries have more cytoreductive surgery and less liver and lung resections than left colon or rectal cancers, and have worse survival after metastasectomy or systemic treatment. Left colon cancers have most liver resections and rectal cancers most lung resections, both with excellent outcomes. Rectal cancer patients seem to suffer from symptoms impairing quality of life caused by the primary tumor itself or the local treatments, while patients with right colon primaries suffer from symptoms caused by the metastatic spread. Thus, right colon, left colon, and rectal cancers are separate disease entities.

## Figures and Tables

**Table 1:** Baseline demographics by primary tumor location.

		Total		Right Colon		Left Colon		Rectum		*p*-Value *
		1080	100%	310	29%	396	37%	374	35%	
Median age (range)		66 (21–90)		68 (21–90)		66 (33–86)		66 (29–89)		0.017
Age groups	≤70 years	711	66%	190	61%	277	70%	244	65%	0.053
	>70 years	369	34%	120	39%	119	30%	130	35%	
Sex	Male	654	61%	163	53%	246	62%	245	66%	0.002
	Female	426	39%	147	47%	150	38%	129	34%	
ECOG performance	0	294	27%	79	25%	120	30%	95	25%	0.388
status	1	598	55%	172	55%	216	55%	210	56%	
	2–3	188	17%	59	19%	60	15%	69	18%	
Primary resection	Right colectomy	42	4%	227	73%	0	0%	0	0%	
	(Sub)Total colectomy	55	5%	16	5%	24	6%	2	1%	
	Left colectomy, Sigma, Hartmann	202	19%	0	0%	266	67%	17	5%	
	Anterior resection	283	26%	0	0%	30	8%	172	46%	
	Abdominoperineal resection	252	23%	0	0%	0	0%	55	15%	
	Other	19	2%	12	4%	4	1%	3	1%	
	Never surgery	227	21%	55	18%	72	18%	125	33%	
Histology	Adenocarcinoma	963	89%	248	80%	369	93%	346	93%	<0.001
	Mucinous adenocarcinoma	107	10%	56	18%	25	6%	26	7%	
	Signet cell carcinoma	6	1%	3	1%	1	0.3%	2	1%	
	MINEN	4	0.4%	3	1%	1	0.3%	0	0%	
Tumor grade	Low	717	81%	185	71%	295	87%	237	83%	<0.001
	High	168	19%	75	29%	43	13%	50	17%	
	Not available	195	-	50	-	58	-	87	-	-
Presentation of	Synchronous	732	68%	222	72%	268	68%	242	65%	0.157
metastases	Metachronous	348	32%	88	28%	128	32%	132	35%	
Number of	1	582	54%	161	52%	225	57%	196	52%	0.122
metastatic sites	2	317	29%	84	27%	116	29%	117	31%	
	3+	181	17%	65	21%	55	14%	61	16%	
Metastatic sites	Liver	809	75%	219	71%	322	81%	268	72%	0.001
	Lung	331	31%	69	22%	97	24%	165	44%	<0.001
	Lymph nodes	272	25%	95	31%	72	18%	105	28%	<0.001
	Peritoneum	170	16%	85	27%	64	16%	21	6%	<0.001
	Local relapse	67	6%	21	7%	19	5%	27	7%	0.336
	Ovarian	26	2%	15	5%	7	2%	4	1%	0.003
	Bone	26	2%	8	3%	6	2%	12	3%	0.301
	Adrenal	15	1%	3	1%	6	2%	6	2%	0.750
	Brain	3	0%	1	0%	2	1%	0	0%	0.406
	Other	89	8%	32	10%	34	9%	23	6%	0.135
Smoking status	Former or never	662	86%	199	88%	252	90%	211	81%	0.006
	Current	106	14%	28	12%	28	10%	50	19%	
	Not available	312	-	83	-	116	-	113	-	-
Mutation status	*RAS* & *BRAF* wt	354	35%	56	19%	165	44%	133	38%	<0.001
	*RAS* mt	553	55%	163	56%	190	51%	200	58%	
	*BRAF*-V600E mt	99	10%	70	24%	16	4%	13	4%	
	(*K*)*RAS* wt	59	-	16	-	21	-	22	-	-
	Not tested	15	-	5	-	4	-	6	-	-
MMR-status	pMMR	410	97%	107	92%	165	97%	138	100%	0.002
	dMMR	14	3%	9	8%	5	3%	0	0%	
	Not available	656	-	194	-	226	-	236	-	-

dMMR = deficient mismatch repair; MINEN = mixed neuroendocrine non-neuroendocrine neoplasms; MMR = mismatch repair; pMMR = proficient mismatch repair. * Crosstabs were calculated for right colon vs. left colon vs. rectum and all alternatives with percentages presented for each demographic factor.

**Table 2:** Resectability and resections and/or local ablative therapy (LAT) according to PTL.

		All Patients		Right Colon		Left Colon		Rectum		*p*-Value *
		1080	100%	310	29%	396	37%	374	35%	
Upfront resectability	Upfront resectable	309	29%	79	26%	127	32%	103	28%	0.008
by central assessment	Borderline resectable	179	17%	44	14%	80	20%	55	15%	
	Non-resectable	592	55%	187	60%	189	48%	216	58%	
Final resectability	Upfront resectable	309	29%	79	26%	127	32%	103	28%	<0.001
status	Converted resectable	137	13%	25	8%	69	17%	43	12%	
	Unconvertable	51	5%	19	6%	18	5%	14	4%	
	Nonresectable mets	583	54%	187	60%	182	46%	214	57%	
Treatment groups	R0–1 resection	326	30%	73	24%	142	36%	111	30%	0.001
	R2-resection or LAT	71	7%	11	4%	34	9%	26	7%	
	Systemic only	660	61%	216	70%	218	55%	226	60%	
	Best supportive care	23	2%	10	3%	2	1%	11	3%	-
Metastasectomies	All patients	399	37%	86	28%	176	44%	137	37%	<0.001
and/or LAT	Single site metastases	309/582	53%	64/161	40%	141/225	63%	104/196	53%	<0.001
	Multiple metastatic sites	90/498	18%	22/149	15%	35/171	21%	33/178	19%	0.409
	Liver procedure	316	29%	57	18%	151	38%	108	29%	<0.001
	Baseline liver mets	310/809	38%	57/218	26%	147/321	46%	106/270	39%	<0.001
	Baseline liver only	266/699	38%	47/195	24%	134/269	50%	85/235	36%	<0.001
	Lung procedures	81	8%	10	3%	30	8%	41	11%	0.001
	Baseline lung mets	46/330	14%	6/68	9%	12/97	12%	28/165	17%	0.229
	Baseline lung only	27/66	41%	4/10	40%	6/12	50%	17/44	39%	0.776
	Cytoreductive surgery	48	4%	22	7%	21	5%	5	1%	0.001
	Baseline peritoneal mets	34/172	20%	17/87	20%	15/64	23%	2/21	10%	0.276
	Baseline peritoneal only	11/43	26%	8/27	30%	2/13	15%	1/3	33%	0.307
	Local relapse resected	41	4%	11	4%	16	4%	14	4%	0.942
	Distant lymphadenectomy	15	1%	6	2%	5	1%	4	1%	0.606
	Gynecologic resection	17	2%	7	2%	9	2%	1	0%	0.043
	Urologic resection	10	1%	3	1%	4	1%	3	1%	0.952
	Subcutaneous resection	10	1%	7	2%	2	1%	1	0%	0.014

Mets = metastases; LAT = local ablative therapy. * Crosstabs was calculated for right colon vs. left colon vs. rectum and all alternatives with percentages presented for each demographic factor.

**Table 3:** Treatment and response to treatment according to primary tumor location.

		Total		Right Colon		Left Colon		Rectum		*p*-Value *
		1080	100%	310	29%	396	37%	374	35%	
Type of treatment	Systemic therapy only	660	61%	216	70%	218	55%	226	60%	<0.001
	Metastasectomy and/or LAT	397	37%	84	27%	176	44%	137	37%	
	Best supportive care	23	2%	10	3%	2	1%	11	3%	
Chemotherapy	Given in any line or intent	1052	100%	299	100%	391	100%	362	100%	-
Number of lines	1	408	39%	124	41%	146	37%	138	38%	0.484
	2	269	26%	81	27%	96	25%	92	25%	
	≥3	375	36%	94	31%	149	38%	132	36%	
First-line chemotherapy	Fluoropyrimidine	1042	99%	295	99%	389	99%	358	99%	0.504
	Oxaliplatin	649	62%	199	67%	239	61%	211	58%	0.090
	Irinotecan	273	26%	61	20%	116	30%	96	27%	0.022
	Bevacizumab	614	58%	198	66%	213	54%	203	56%	0.005
	EGFR-inhibitor	148	14%	15	5%	72	18%	61	17%	<0.001
Best response in first line	PR/CR/NED	641	62%	159	54%	257	67%	225	64%	<0.001
	SD	292	28%	88	30%	106	27%	98	28%	
	PD	99	10%	46	16%	23	6%	30	8%	
	Not available	20	-	6	-	5	-	9	-	-
Chemotherapy all lines	Fluoropyrimidine	1045	99%	296	99%	390	100%	359	99%	0.437
	Oxaliplatin	836	79%	241	81%	315	81%	280	77%	0.468
	Irinotecan	763	73%	206	69%	295	75%	262	72%	0.161
	VEGF-inhibitor	756	72%	227	76%	273	70%	256	71%	0.176
	EGFR-inhibitor	313	30%	46	15%	147	38%	120	33%	<0.001

CR = complete response; LAT = local ablative therapy; NED = no evidence of disease; PD = progressive disease; PR = partial response; SD = stable disease. * Crosstabs were calculated for right colon vs. left colon vs. rectum and all alternatives with percentages presented for each demographic factor.

**Table 4:** Univariable and multivariable Cox regression model for overall survival.

			Univariable			Multivariable		
		*N*	HR	95% CI	*p*-Value	HR	95% CI	*p*-Value
Age continuous (years)		1080	1.02	1.01–1.02	<0.001	1.00	0.99–1.00	0.287
Primary tumor location	Right colon	310	1			1		
	Left colon	396	0.57	0.47–0.68	<0.001	0.77	0.63–0.93	0.007
	Rectum	374	0.61	0.51–0.73	<0.001	0.64	0–53–0.78	<0.001
Number of metastatic	1	582	1			1		
sites	2	317	1.86	1.58–2.19	<0.001	1.31	1.11–1.56	0.002
	3–5	181	2.62	2.16–3.17	<0.001	1.78	1.46–2.17	<0.001
ECOG Performance status	0	294	1			1		
	1	598	1.77	1.47–2.13	<0.001	1.48	1.23–1.79	<0.001
	2–3	188	3.72	2.98–4.65	<0.001	2.56	2.03–3.23	<0.001
Type of treatment	Systemic therapy only	660	1			1		
	Metastasectomy and/or LAT	397	0.19	0.16–0.23	<0.001	0.24	0.20–0.29	<0.001
	Best supportive care	23	10.52	6.86–16.12	<0.001	11.02	6.89–17.63	<0.001
Mutation groups	*RAS* & *BRAF* wt	354	1			1		
	*RAS* mt	553	1.46	1.23–1.73	<0.001	1.41	1.19–1.68	<0.001
	*BRAF*-V600E mt	99	3.13	2.42–4.04	<0.001	2.03	1.54–2.69	<0.001
	(*K*)*RAS* wt	59	2.96	2.19–4.00	<0.001	2.37	1.74–3.24	<0.001
	Not tested	15	2.48	1.38–4.44	0.002	2.56	1.40–4.68	0.002
MMR–status	pMMR	410	1			1		
	dMMR	14	1.06	0.55–2.07	0.857	0.69	0.35–1.36	0.287
	Not tested	656	1.35	1.16–1.57	<0.001	0.98	0.83–1.15	0.768

dMMR = deficient mismatch repair; ECOG = European Cooperative Oncology Group; LAT = local ablative therapy; MMR = mismatch repair; pMMR = proficient mismatch repair.

**Table 5:** Comparisons of index measures between primary tumor location in different treatment phases.

	15D		EQ-5D		VAS		GHS		Symptom Burden *		Functioning Scale Sum	
	∆	*p* Value	∆	*p* Value	∆	*p* Value	∆	*p* Value	∆	*p* Value	∆	*p* Value
**Right colon vs. Left colon**												
Post-resection	0.009	0.794	−0.074	0.312	−3.6	0.565	−1.0	0.943	104	0.263	−29	0.494
Remission	0.010	0.499	0.000	0.752	−3.0	0.303	0.4	0.853	23	0.753	0	0.848
Systemic therapy	0.001	0.533	0.001	0.570	−1.7	0.273	−3.6	0.232	−9	0.794	−31	0.190
Best supportive care	**−0.069**	0.237	**−0.108**	0.197	0.8	0.877	**−12.2**	0.197	60	0.400	−82	0.255
**Right colon vs. Rectum**												
Post-resection	**−0.053**	0.169	**−0.140**	0.069	**−10.5**	**0.044**	**−5.4**	0.627	**142**	0.140	**−104**	0.077
Remission	**0.020**	0.512	−0.010	0.519	−2.2	0.638	0.8	0.768	−59	0.217	19	0.798
Systemic therapy	0.000	0.642	−0.010	0.689	0.1	0.993	−2.0	0.564	−49	0.172	−52	**0.023**
Best supportive care	0.007	0.837	0.013	0.902	−2.2	0.930	**−9.4**	0.299	−38	0.837	**−125**	0.142
**Left colon vs. Rectum**												
Post-resection	**−0.062**	**0.012**	**−0.066**	**0.050**	−6.9	0.157	−4.5	0.370	38	0.455	−75	**0.024**
Remission	0.010	0.904	−0.010	0.688	0.8	0.628	0.3	0.741	−82	0.072	19	0.603
Systemic therapy	0.010	0.823	−0.000	0.733	1.7	0.346	1.6	0.433	−40	0.259	−21	0.310
Best supportive care	**0.076**	0.217	0.121	0.462	−3.0	0.531	2.8	0.742	−98	0.538	**143**	0.810

∆ = Difference between means of the two primary tumor locations (first minus second). Minimal clinically important difference (MID) and statistical differences are bolded: 15D (range 0–1.000): ≥|0.015|; EQ-5D (range 0–1.00): ≥|0.08|; EQ-5D Visual Analogue Scale (VAS; 0–100): ≥|7|; Quality of Life Questionnaire (QLQ)-C30 Global Health Score (GHS, range 0–100) ≥|5|. * Symptom burden sum of 26 symptoms (range 0–2600): ≥|26 × 5|, and functioning scale sum (0–1000): ≥|10 × 5|, of 10 function scales from QLQ-C30 and CR-29.

## Data Availability

The data collected for this study can be made available to others in a de-identified form after all primary and secondary endpoints have been published, in the presence of a data transfer agreement, and if the purpose of use complies with Finnish legislation. Requests for data sharing can be made to the corresponding author, including a proposal that must be approved by the steering committee.

## Supplementary Materials

The following supporting information can be downloaded at: https://www.mdpi.com/article/10.3390/cancers16051052/s1, Table S1. Laboratory findings in right colon, left colon, and rectal primary tumor location; Table S2. Univariable and multivariable Cox regression model for overall survival using only baseline factors; Table S3. Health-related quality of life indexes in different treatment phases according to primary tumor location; Figure S1. The mean 15D dimensions in different treatment phases for right colon, left colon, and rectal primary tumor location.
