# Supplementary material for: Impact of Primary Tumor Location on Demographics, Resectability, Outcomes, and Quality of Life in Finnish Metastatic Colorectal Cancer Patients (Subgroup Analysis of the RAXO Study)
Source: Cancers (Basel). 2024 Mar 5;16(5):1052. doi: 10.3390/cancers16051052 (PMC10931274; doi:10.3390/cancers16051052)
Supplement: Supplementary file 1 [file cancers-16-01052-s001.zip › cancers-2887814-supplementary.pdf]

**Table S1**. Laboratory findings in right colon, left colon and rectal primary tumor location

| | | Total | | Right colon | | Left colon | | Rectum | | P-value* |
|---|---|---|---|---|---|---|---|---|---|---|
| | | N=1080 | 100 % | 310 | 29 % | 396 | 37 % | 374 | 35 % | |
| Haemoglobin | ≥11 g/dL | 917 | 83 % | 233 | 74 % | 344 | 86 % | 340 | 87 % | <0.001 |
| | <11 g/dL | 193 | 17 % | 84 | 26 % | 58 | 14 % | 51 | 13 % | |
| Leucocytes | ≤10 ^9/L | 919 | 83 % | 261 | 82 % | 332 | 83 % | 326 | 83 % | 0.927 |
| | >10 ^9/L | 191 | 17 % | 56 | 18 % | 70 | 17 % | 65 | 17 % | |
| Thrombocytes | ≤400 ^9/L | 813 | 73 % | 227 | 72 % | 287 | 71 % | 299 | 76 % | 0.201 |
| | >400 ^9/L | 297 | 27 % | 90 | 28 % | 115 | 29 % | 92 | 24 % | |
| Neutrophils | ≤6.7 ^9/L | 836 | 84 % | 244 | 85 % | 305 | 83 % | 287 | 83 % | 0.726 |
| | >6.7 ^9/L | 164 | 16 % | 43 | 15 % | 61 | 17 % | 60 | 17 % | |
| C-reactive protein | ≤10 mg/L | 489 | 54 % | 149 | 56 % | 170 | 52 % | 170 | 54 % | 0.581 |
| | >10 mg/L | 417 | 46 % | 117 | 44 % | 158 | 48 % | 142 | 46 % | |
| Albumin | ≥30 g/L | 544 | 84 % | 166 | 83 % | 206 | 84 % | 172 | 83 % | 0.922 |
| | <30 g/L | 106 | 16 % | 33 | 17 % | 38 | 16 % | 35 | 17 % | |
| Estimated glomerular | ≥90 ml/min/1.73m2 | 484 | 44 % | 126 | 40 % | 194 | 49 % | 164 | 43 % | 0.076 |
| filtration rate | 60-89 ml/min/1.73m2 | 433 | 40 % | 123 | 39 % | 145 | 37 % | 165 | 43 % | |
| | 30-59 ml/min/1.73m2 | 170 | 16 % | 63 | 20 % | 54 | 14 % | 53 | 14 % | |
| | <30 ml/min/1.73m2 | 5 | 0 % | 1 | 0 % | 2 | 1 % | 2 | 1 % | |
| Alkaline phosphatase | ≤105 U/L | 720 | 66 % | 201 | 64 % | 249 | 62 % | 270 | 70 % | 0.065 |
| | >105 U/L | 379 | 34 % | 112 | 36 % | 151 | 38 % | 116 | 30 % | |
| Alanine amino transferase | Under upper limit of normal | 895 | 83 % | 253 | 82 % | 325 | 82 % | 317 | 84 % | 0.600 |
| | Over upper limit of normal | 187 | 17 % | 56 | 18 % | 72 | 18 % | 59 | 16 % | |
| Hyperbilirubinemia | ≤25 μmol/L | 884 | 85 % | 247 | 83 % | 329 | 86 % | 308 | 85 % | 0.671 |
| | >25 μmol/L | 157 | 15 % | 49 | 17 % | 54 | 14 % | 54 | 15 % | |
| Carcinoembyonic antigen | ≤5 μ/L | 316 | 29 % | 102 | 33 % | 105 | 27 % | 109 | 29 % | 0.194 |
| | >5 μ/L | 773 | 71 % | 210 | 67 % | 291 | 73 % | 272 | 71 % | |
| Cancer antigen 19-9 | ≤26 kU/L | 297 | 46 % | 85 | 42 % | 102 | 44 % | 110 | 51 % | 0.134 |
| | >26 kU/L | 350 | 54 % | 116 | 58 % | 130 | 56 % | 104 | 49 % | |

* Crosstabs was calculated for right colon vs left colon vs rectum and all alternatives with percentages presented for each demographic factor

**Table S2**. Univariable and multivariable Cox regression model for overall survival using only baseline factors

| | | | Univariable | | | Multivariable | | |
|---|---|---|---|---|---|---|---|---|
| | | N | HR | 95% CI | p-value | HR | 95% CI | p-value |
| Age | | 1080 | 1.02 | 1.01-1.02 | <0.001 | 1.00 | 0.99-1.01 | 0.616 |
| Primary tumor location | Right colon | 310 | 1 | | | 1 | | |
| | Left colon | 396 | 0.57 | 0.47-0.68 | <0.001 | 0.69 | 0.57-0.83 | <0.001 |
| | Rectum | 374 | 0.61 | 0.51-0.73 | <0.001 | 0.63 | 0.51-0.76 | <0.001 |
| Number of metastatic sites | 1 | 582 | 1 | | | 1 | | |
| | 2 | 317 | 1.86 | 1.58-2.19 | <0.001 | 1.23 | 1.04-1.47 | 0.018 |
| | 3-5 | 181 | 2.62 | 2.16-3.17 | <0.001 | 1.62 | 1.32-1.97 | <0.001 |
| ECOG Performance status | 0 | 294 | 1 | | | 1 | | |
| | 1 | 598 | 1.77 | 1.47-2.13 | <0.001 | 1.56 | 1.30-1.88 | <0.001 |
| | 2-3 | 188 | 3.72 | 2.98-4.65 | <0.001 | 2.78 | 2.20-3.50 | <0.001 |
| Upfront resectability | Upfront resectable | 309 | 1 | | | 1 | | |
| | Borderline resectable | 179 | 1.43 | 1.11.-1.86 | <0.001 | 1.29 | 0.99-1.68 | 0.058 |
| | Un-resectable | 592 | 4.65 | 3.82-5.67 | <0.001 | 3.53 | 2.85-4.37 | <0.001 |
| Mutation groups | *RAS*&*BRAF* wt | 354 | 1 | | | 1 | | |
| | *RAS* mt | 553 | 1.46 | 1.23-1.73 | <0.001 | 1.22 | 1.03-1.45 | 0.021 |
| | *BRAF*-V600E mt | 99 | 3.13 | 2.42-4.04 | <0.001 | 1.85 | 1.44-2.39 | <0.001 |
| | (*K*)*RAS* wt | 59 | 2.96 | 2.19-4.00 | <0.001 | 2.01 | 1.50-2.69 | <0.001 |
| | Not tested | 15 | 2.48 | 1.38-4.44 | 0.002 | 2.38 | 1.32-4.28 | 0.004 |
| MMR-status | pMMR | 410 | 1 | | | 1 | | |
| | dMMR | 14 | 1.06 | 0.55-2.07 | 0.857 | 0.86 | 0.46-1.59 | 0.620 |
| | Not tested | 656 | 1.35 | 1.16-1.57 | <0.001 | 1.08 | 0.93-1.27 | 0.321 |

dMMR=deficient mismatch repair, ECOG=European Cooperative Oncology Group, LAT=local ablative therapy, MMR=mismatch repair, pMMR=proficient mismatch repair

**Table S3**. Health-related quality of life indexes in different treatment phases according to primary tumor location

| | | Right colon | | Left colon | | Rectum | |
|---|---|---|---|---|---|---|---|
| | | Mean | SD | Mean | SD | Mean | SD |
| Post-resection | 15D index | 0.86 | 0.10 | 0.85 | 0.10 | 0.91 | 0.06 |
| | EQ-5D index | 0.76 | 0.20 | 0.83 | 0.13 | 0.90 | 0.13 |
| | EQ-5D VAS | 69 | 15 | 73 | 16 | 80 | 10 |
| | Global health status | 73 | 22 | 74 | 20 | 78 | 15 |
| Remission | 15D index | 0.91 | 0.08 | 0.90 | 0.08 | 0.89 | 0.11 |
| | EQ-5D index | 0.87 | 0.13 | 0.87 | 0.13 | 0.88 | 0.13 |
| | EQ-5D VAS | 76 | 15 | 79 | 14 | 79 | 13 |
| | Global health status | 77 | 16 | 77 | 19 | 76 | 17 |
| Systemic therapy | 15D index | 0.86 | 0.09 | 0.87 | 0.09 | 0.86 | 0.09 |
| | EQ-5D index | 0.80 | 0.17 | 0.81 | 0.16 | 0.81 | 0.16 |
| | EQ-5D VAS | 69 | 15 | 71 | 16 | 69 | 15 |
| | Global health status | 66 | 20 | 69 | 16 | 68 | 17 |
| Best supportive care | 15D index | 0.73 | 0.10 | 0.80 | 0.12 | 0.72 | 0.13 |
| | EQ-5D index | 0.61 | 0.28 | 0.71 | 0.21 | 0.59 | 0.35 |
| | EQ-5D VAS | 55 | 21 | 55 | 18 | 58 | 19 |
| | Global health status | 45 | 25 | 57 | 20 | 55 | 19 |

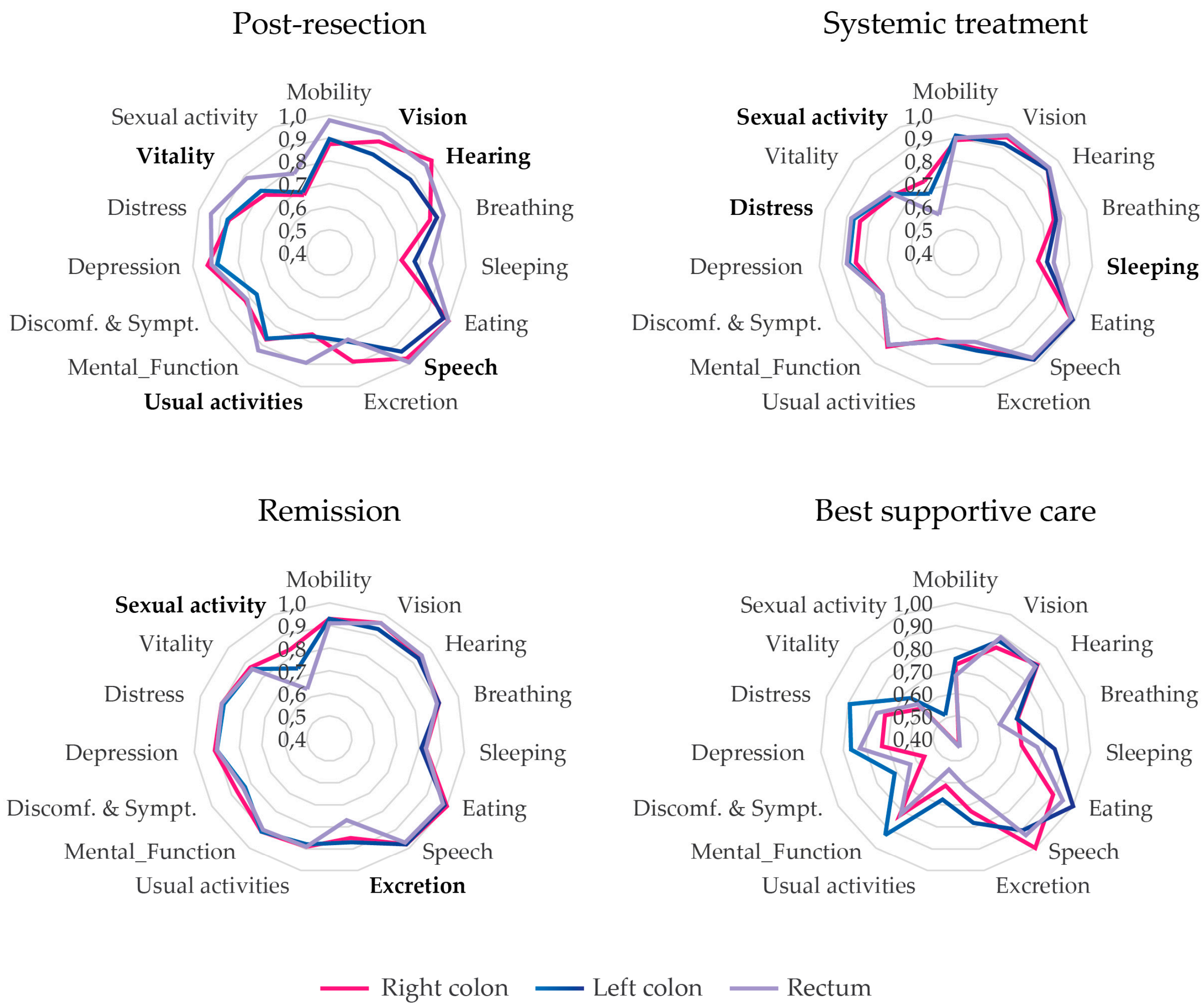


**Figure S1**. The mean 15D dimensions in different treatment phases for right colon, left colon, and rectal primary tumor location
